# Source analysis and ecological risk assessment of heavy metals in farmland soils around heavy metal industry in Anxin County

**DOI:** 10.1038/s41598-022-13977-6

**Published:** 2022-06-22

**Authors:** Guoliang Zhao, Ye Ma, Yuzhen Liu, Jiemin Cheng, Xiaofeng Wang

**Affiliations:** College of Geography and Environment, Shandong Normal University, Jinan, 250014 China

**Keywords:** Environmental social sciences, Environmental sciences, Environmental chemistry, Environmental impact

## Abstract

Studying the pollution status, spatial distribution characteristics, and sources of heavy metals in farmland soil in Anxin County will provide a method basis for the next step of soil remediation. This study investigates the contents of Zn, Cu, Pb, Cd, and Ni in wheat grains and soil samples. Moreover, different methods are used to evaluate soil heavy metal pollution. The results show that the soil in the study area is weakly alkaline. Cu, Zn, and Ni contents in the ground are lower than the risk screening values for soil contamination of agricultural land. In comparison, Cd and Pb contents are higher than the screening value of soil pollution risk of agricultural land, and the proportion of points lower than the control value of soil pollution risk of agricultural land are 64.58% and 16.67%, respectively. The farmland with high Cd and Pb content is mainly distributed near roads and factories and concentrated primarily on 0-20 cm topsoil. The Cd content in wheat grains meets the standard, but 4.17% of the samples are close to 0.1 mg kg^−1^ (more than 0.09 mg kg^−1^). The Pb content of 50% of the wheat grain samples exceeds the lead limit in the standard. The evaluation results of the single factor pollution index and geoaccumulation index show that the pollution degree of heavy metals in the soil is Cd > Pb > Cu > Zn > Ni. The potential ecological risk index in the study area is 288.83, and the soil heavy metal pollution is at a moderate-considerable ecological risk level. The average value of Cd's single-factor environmental risk index is 233.51, which belongs to the high environmental risk and is the main influencing factor. Cd and Pb in soil are significantly disturbed by the production activities of heavy metal processing enterprises around the farmland. It is speculated that there are two primary sources of soil heavy metal pollution in the study area. Cd, Pb, Zn, and Cu are mainly industrial and mobile sources, and Ni is primarily agricultural and natural sources.

## Introduction

Soil is a necessary guarantee for the survival and development of human society. The soil environmental quality of cultivated land directly affects food security and the quality of agricultural products and is closely related to people's health and social development. With the continuous development of the economy and society, human activities have led to increasing pollution of heavy metals in the soil^1^. Heavy metal pollution in the soil can lead to the destruction of the balance of the ecosystem. Some crops cannot grow and develop normally, resulting in reduced agricultural output and a decline in quality. At the same time, it enters the human body through the food chain and other channels and accumulates in the human body, endangering human health^2–4^.

With the development of industry, more and more pollutants enter farmland soil through various ways, such as atmospheric settlement^5^, sewage irrigation^6,7^, agricultural production^8^, mining^9^. Farmland around heavy metal enterprises is threatened by heavy metal pollution^10^. A nationwide survey in China revealed that little pollution was found in typical farmland, far from noticeable anthropogenic emissions, but Cd and Hg in mining-smelting and industrial areas significantly accumulated in soil^11^.

There is a consensus that heavy metals have great harm to the ecological environment^12^. The effects of heavy metals in soil on plants, soil microorganisms, and soil animals have been well documented^13^. The excessive accumulation of heavy metals in soils has led to phytotoxic metabolism and inevitably poses risks to human health via the food chain^14–16^. Long-term exposure to heavy metals contributed to mental and behavioral disorders and increased the risk of cancer^17^. Therefore, it is necessary to conduct soil heavy metal pollution assessment.

The single factor index method and Nemerow pollution index method often are used to assess soil heavy metals pollution^18^. Geoaccumulation index method combines the changes of background value caused by natural diagenesis in the evaluation of soil heavy metal pollution^19^, the potential ecological risk index method comprehensively considers the toxic effects of heavy metals^20^, these two methods have been widely used in the evaluation of soil pollution. Many research scholars have also studied the bioavailability of heavy metals^21^. The existing soil pollution evaluation methods are limited to whether the content exceeds the standard, and there are few studies on the analysis of the background value of soil elements and the source analysis of heavy metals. There is no comprehensive evaluation of soil heavy metal pollution.

On April 1, 2017, China's State Council decided to establish Xiong’an New Area in Hebei Province of North China. The planning scope of Xiong’an New Area covers Xiong County, Rongcheng County, Anxin County, and some surrounding areas in Hebei Province of China. It is located in the hinterland of Beijing, Tianjin, and Baoding City. It is 105 km away from Beijing. Anxin County is the most extensive footwear production base, waste metal distribution center, and distribution center in North China. Metal smelting enterprises mainly focus on lead-containing non-ferrous metal smelting. Due to the lack of unified planning and management, they are mainly small enterprises, and the distribution is relatively scattered. Some enterprises still have high energy consumption, backward production technology, and low comprehensive utilization rate^22^. Extensive development will significantly impact the soil environment^23–25^. So far, few articles have conducted comprehensive research on farmland soil ecological environment in Anxin County, and the risk of heavy metal pollution is unknown. On December 25, 2018, the State Council of China issued and implemented the *Reply of the State Council on the General Planning of the Hebei Xiong’an New District (2018–2035)*, requiring effective control of rural non-point source pollution. Studying the pollution situation, spatial distribution characteristics, and sources of heavy metals in farmland soil in Anxin County will provide a method basis for the next step of soil remediation.

## Materials and methods

### General situation of research district

**Figure 1:**
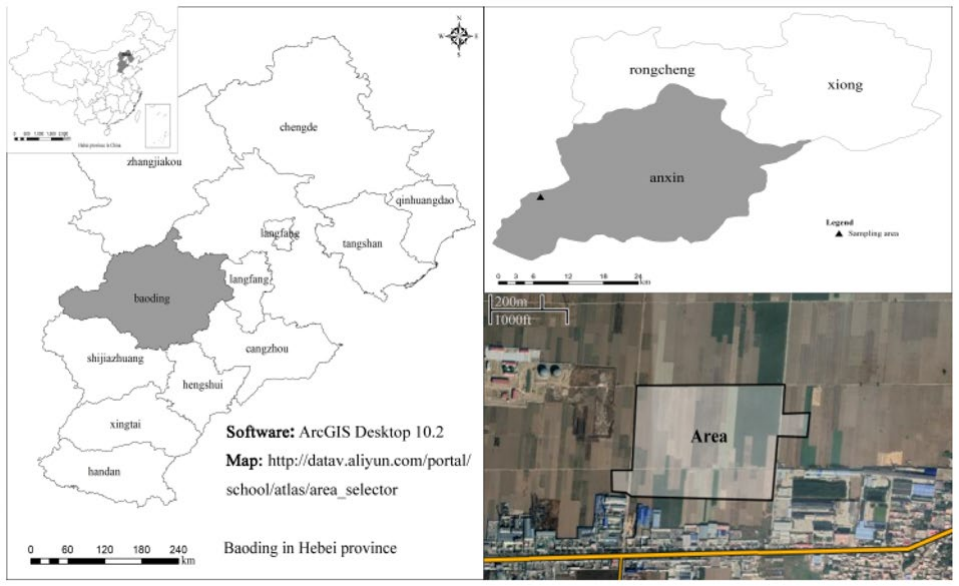
Geographical location map of research district.

Anxin County is located in the central part of Hebei Province (Fig. 1), with 738.6 square kilometers. There is an alluvial depression plain in the northwest and north and the largest freshwater lake in the North China Plain—Baiyangdian, in the east, with abundant aquatic animal and plant resources. The total terrain is slightly inclined from the northwest to the southeast, the terrain is flat, and the natural slope of the ground is 1:2000. It is a gently inclined plane with a deep soil layer, open terrain, and low vegetation coverage. Anxin County is located in the mid-latitude zone, a warm temperate monsoon continental climate with four distinct seasons, with an average annual temperature of 11.7 °C and average annual precipitation of 522.9 mm. There are mineral resources such as oil, natural gas, geothermal energy, clay, and sand in Anxin County.

According to the seventh census data, as of 0:00 on November 1, 2020, the permanent population of Anxin County was 453,723^26^. The county's arable land area is 480,000 mu, the soil with higher terrain develops into cinnamon soil, and the soil with lower terrain develops into flavor-aquic soil^27^. The main crops are wheat and corn, which are ripened twice a year. Anxin County is the most extensive footwear production base, waste metal distribution center, and distribution center in North China. Overall, industrial development is lower, and the ecological environment is better in the region. The main survey area of this study is the farmland near the heavy metal enterprise in Anxin County, with a total survey area of about 200 mu.

### Collection of samples

**Figure 2:**
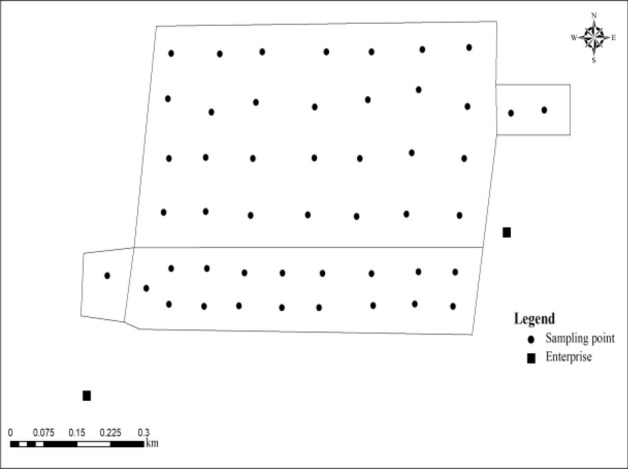
Location of sampling points in Anxin County.

Referring to the *Technical rules for monitoring of environmental quality of farmland soil* (NY/T 395-2012)^28^, the study area was divided into cells of 50 m × 50 m, and soil samples in the plow layer (0–20 cm) were collected in the cells. 48 soil samples were collected by the plum-shaped distribution method (Fig. 2). The soil samples were flattened and placed on the laboratory balcony to dry naturally. All the small gravels and other debris in the soil were picked out. After grinding, they passed through a 0.84 mm nylon sieve, and then a part was taken and ground through a 0.15 mm nylon sieve. Pack in a ziplock bag and store in a desiccator for later use.

Four soil profile sampling points were selected, D0 (38.8849 N, 115.9112E), D1 (38.8362 N, 115.7078E), D2 (38.8353 N, 115.7081E), D3 (38.8345 N, 115.7081E). D0 was no heavy metal-contaminated farmland soil in the previous survey, and D1, D2, and D3 were heavy metal-contaminated farmland soil in the study area. Dig a soil profile with a length × width × depth of 2.0 m × 1.0 m × 1.5 m in the soil layers of 0–20 cm, 20–40 cm, 40–60 cm, 60–80 cm, and 80–100 cm sampling. Sample preparation was as described above.

The wheat sampling points corresponded to the soil sampling points and were collected by the plum-shaped distribution method one week before the wheat harvest. A total of 48 wheat samples were collected. After the collected wheat ears were placed on the balcony to dry naturally, the outer skin was manually rubbed to obtain the grain part, which was pulverized with a pulverizer, passed through a 0.15 mm nylon sieve, packaged in a self-sealing bag, and placed in a desiccator for storage. In this study, 48 soil and 48 wheat grain samples were collected in the study area on July 10, 2019 (Fig. 2).

### Basic properties of the soil

The air-dried soil samples sieved through 0.84 mm were tested for pH (The soil samples were extracted with 0.01 mol L^−1^ CaCl_2_ solution according to the water-soil ratio of 2.5:1. After stirring, let it stand for 30 min, and use the DZS-706 pH meter of Shanghai Yidian Scientific Instrument Co., Ltd. to measure.) and available heavy metals (by DTPA [pH = 7.3] extraction)^29^. The air-dried soil samples sieved through 0.15 mm were tested for total Cu, Zn, Pb, Cd, and Ni (HCl-HNO_3_-HClO_4_-HF digestion). Put 0.1 g of air-dried soil samples sieved with 0.15 mm into a digestion tube, and add a few drops of water to moisten. Then add 3 mL of HCl and 1 mL of HNO_3_, cover with a small funnel, and place it in a fume hood to soak overnight. The next day, it was put into the SH220F graphite digestion apparatus, and the temperature was gradually raised to 130 °C for digestion for 2 h. When the remaining 2–3 mL was evaporated, it was removed and placed in a ventilated place to cool. Add 1 mL of HClO_4_ and 2 mL of HF, cover with a small funnel, heat up to 160 °C in steps, continue to digest for 2 h, then open the lid and heat to remove silicon. When heated until white smoke of perchloric acid appears, cover it to make the black organic substances on the inner wall of the digestion tube disappear, open the cover, and steam until the content is viscous but not flowing, then remove and cool. After cooling completely, rinse the inner wall and lid with deionized water, transfer the total amount and make up the volume to a 50 mL volumetric flask. The heavy metal concentrations on the filter were analyzed using the HK-8100 inductively coupled plasma emission spectrum and TAS-990AFG atomic absorption spectrophotometer.

### Absorption of heavy metals in wheat

0.5–1.0 g of dry-milled wheat grain sample was placed in a conical beaker, soaked in concentrated nitric acid (25 mL), and kept overnight (∼18 h). Afterward, the beaker was placed on a hot plate and heated at 100 °C for 0.5 h. After cooling down, the sample was extracted with perchloric acid (5 mL); the heating process continued until the solution was colorless, transparent, and evaporated to a final volume of 2 mL. Finally, two drops of concentrated nitric acid were added to the solutions. The heavy metal content in the samples was determined using the HK-8100 inductively coupled plasma emission spectrum and TAS-990AFG atomic absorption spectrophotometer.

### Sample analysis method

Heavy metals in soil samples are determined by *Soil quality-Analysis of soil heavy metals-atomic absorption spectrometry with aqua regia digestion* (NY/T 1613–2008)^30^, and available Pb and Cd by *Soil quality-Analysis of available lead and cadmium contents in soils-Atomic absorption spectrometry* (GB/T 23769-2009)^31^ for determination, the detection limit is Cu 2 mg kg^−1^, Zn 0.4 mg kg^−1^, Pb 5 mg kg^−1^, Cd 0.01 mg kg^−1^, Ni 2 mg kg^−1^. Cd in wheat samples is determined according to the *National Food Safety Standard-Determination of Cadmium in Food* (GB 5009.15–2014)^32^, the detection limit is 0.001 mg kg^−1^, and the quantification limit is 0.003 mg kg^−1^. Pb in wheat samples is determined according to the *National Food Safety Standard-Determination of Lead in Food* (GB 5009.12-2017)^33^ graphite furnace atomic absorption spectrometry, the detection limit is 0.02 mg kg^−1^, and the quantification limit is 0.04 mg kg^−1^.

For elemental analysis, three replicate samples, and national first-class reference materials (soil standard sample GBW08303 and shrub dead leaf sample GBW07603) were used for quality monitoring. The quality of sample analysis and test and the detection limit of each element index meet the technical specification requirements for sample analysis of ecological geochemical evaluation^34^. The analytical data's reporting rate, accuracy, and precision reached 100%. The analytical data is reliable.

### Assessment method

#### The single pollution index method

In this study, the single pollution index method was used to evaluate the contamination of heavy metals in soil. The single pollution index method is one of the most widely used methods to determine the degree of pollution at home and abroad, and it can reflect the pollution degree of different pollutants to the environment^35^. The damage degree of heavy metal pollutants in soil was determined by the single factor pollution index method. *Soil environmental quality-Risk control standard for soil contamination of agricultural land (Trial)* (GB15618-2018)^36^ issued by the Ministry of Ecological and Environment of China was as an assessment standard and shown in Table 1. Among them, risk screening values for soil contamination of agricultural land (*A*) means that the pollutant content in the soil of agricultural land is equal to or lower than this value, and the risk to the quality and safety of agricultural products, the growth of crops or the soil ecological environment is low and can be ignored in general. If the value exceeds this, there may be risks to the quality and safety of agricultural products, crop growth, or soil ecological environment, and soil environmental monitoring and coordinated monitoring of agricultural products should be strengthened. In principle, safe use measures should be taken. Risk intervention values for soil contamination of agricultural land (*B*) means that if the content of pollutants in the agricultural land exceeds this value, and the edible agricultural products do not meet the quality and safety standards, the risk of agricultural land soil pollution is high. In principle, strict control measures should be taken. The pollution index of heavy metals was calculated with Eq. (1):

**Table 1:** Risk control standard value for soil contamination of agricultural land (mg kg^−1^).

Risk degree	Cu	Zn	Pb	Cd	Ni
*A*	100	300	170	0.6	190
*B*	–	–	1000	4	–

1 $${P}_{i}=\frac{{C}_{i}}{{S}_{i}}$$

where, *C*_*i*_ is the total measured amount of heavy metal *i* in soil (mg kg^−1^). *S*_*i*_ is the standard assessment value of heavy metal *i* (mg kg^−1^), the value is risk screening values for soil contamination of agricultural land (*A*) of *Soil environmental quality—Risk control standard for soil contamination of agricultural land (Trial)* (GB15618-2018). *P*_*i*_ is the pollution index of heavy metal *i* in soil. According to the *P*_*i*_ value, when *P*_*i*_ is less than or equal to 1, the soil does not exceed the national standard. 1 < *P*_*i*_ ≤ 2 indicates light pollution, 2 < *P*_*i*_ ≤ 3 indicates moderate pollution, *P*_*i*_ > 3 indicates heavy pollution^37^.

#### The potential ecological risk index method

The potential ecological risk index (RI) was used to evaluate the ecological risk of heavy metals in soil^38^. RI was calculated with Eq. (2):

2 $$RI = \sum\limits_{i = 1}^{m} {E_{r}^{i} = \sum\limits_{i = 1}^{m} {T_{r}^{i} } } \times C_{f}^{i} = \sum\limits_{i = 1}^{m} {T_{r}^{i} } \times \frac{{C^{i} }}{{C_{n}^{i} }}.$$

where, *C*^*i*^ is the total measured amount of heavy metal *i* in soil (mg kg^−1^), *C*_*n*_^*i*^ is the corresponding background value of heavy metal *i* in soil (mg kg^−1^) of Hebei Province^39^. *T*_*r*_^*i*^ is the toxicity coefficient of heavy metal *i* in soil, *E*^*i*^_*r*_ is the single factor ecological risk index of heavy metal *i* in soil, and *RI* is the comprehensive ecological risk index of heavy metal *i* in soil. *T*_*r*_^*i*^ of Cu, Zn, Pb, Cd, and Ni is 5, 1, 5, 30, and 5, respectively^38,40^. The E^i^_r_ and RI values are classified with a system described by Hakanson et al., and the classification is shown in Table 2.

**Table 2:** Classification standard of potential ecological risk.

Risk level	E^i^_r_	Risk degree of E^i^_r_	RI	Risk degree of RI
A	E^i^_r_ < 40	Low	RI < 150	Low
B	40 ≤ E^i^_r_ < 80	Moderate	150 ≤ RI < 300	Moderate
C	80 ≤ E^i^_r_ < 160	Considerable	300 ≤ RI < 600	Considerable
D	160 ≤ E^i^_r_ < 320	High	600 ≤ RI	Very high
E	320 ≤ E^i^_r_	Very high	—	—

#### The geoaccumulation index method

The geoaccumulation index method was proposed by Muller in 1969, which was mainly used to evaluate the pollution degree of heavy metals in sediments^41^. It was calculated with Eq. (3):

3 $${I}_{geo}={\mathrm{log}}_{2}\frac{{C}_{i}}{1.5{B}_{i}}$$

where, *C*_*i*_ is the total measured amount of heavy metal *i* in soil (mg kg^−1^). *B*_*i*_ is to evaluate the geochemical background values of heavy metal *i*. *I*_*geo*_ is the geoaccumulation index of heavy metal *i* in soil. In this study, *B*_*i*_ is selected from the average value of the Chinese soil element background. Table 3 shows the grading of the local accumulation index^42^.

**Table 3:** Standard for evaluation of soil pollution degree by land accumulation index.

Level of pollution	I_geo_	Degree of pollution
A	I_geo_ ≤ 0	Clean
B	0 < I_geo_ ≤ 1	Light pollution
C	1 < I_geo_ ≤ 2	Middling pollution
D	2 < I_geo_ ≤ 3	Moderate pollution
E	3 < I_geo_ ≤ 4	Biased pollution
F	4 < I_geo_ ≤ 5	Heavy pollution
G	5 < I_geo_	Serious pollution

In formula (2) and (3), *C*_*n*_^*i*^ and *B*_*i*_ selected the average value of every single element in layer *A* soil of Hebei Province as the element background value^39^, and the element background values of Cu, Zn, Pb, Cd, and Ni are 21.8 mg kg^−1^, 78.4 mg kg^−1^, 21.5 mg kg^−1^, 0.094 mg kg^−1^, and 30.8 mg kg^−1^, respectively.

#### Transfer coefficient of heavy metals

The element transfer coefficient can reflect the plant's ability to accumulate heavy metals and is the ratio of the content of an element in the plant sample to the content of the corresponding element in the soil. It can, to some extent, explain the enrichment of heavy metals in the plant body^43^. Transfer coefficient was calculated with Eq. (4):

4 $${\text{K}}=\frac{{C}_{p}}{{C}_{s}}$$

where, K is the transfer coefficient of heavy metal *i*, *C*_*p*_ is the content of heavy metal *i* in plant samples (mg kg^−1^), while *C*_*s*_ is the content of heavy metal *i* in soil (mg kg^−1^).

The experimental data analysis was carried out with Microsoft Office Excel 2010 software, ArcGIS Desktop 10.2 was used for Kriging analysis of spatial points, Origin 2017 software was used for mapping, and Adobe Photoshop CC 2018 was used for image merging.

### Ethical approval

All plant samples of this study complied with the *Regulation for the collection of genetic resources* (HJ 628–2011), *The Technical Specification for soil Environmental monitoring* (HJ/T 166–2004), the *IUCN Policy Statement on Research Involving Species at Risk of Extinction* and the *Convention on the Trade in Endangered Species of Wild Fauna and Flora*. We have permission to collect wheat seeds. We guarantee that all samples are used for scientific research only.

## Results and discussion

### Descriptive statistical analysis

#### Basic properties of soil

**Figure 3:**
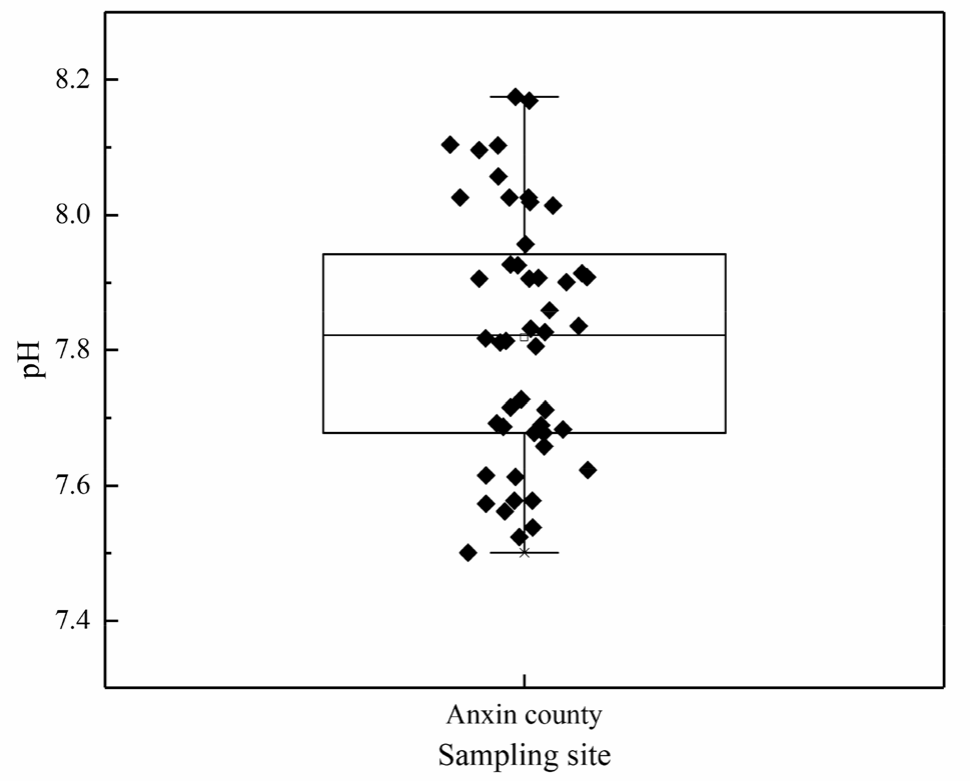
Box-plot of soil pH distribution.

**Figure 4:**
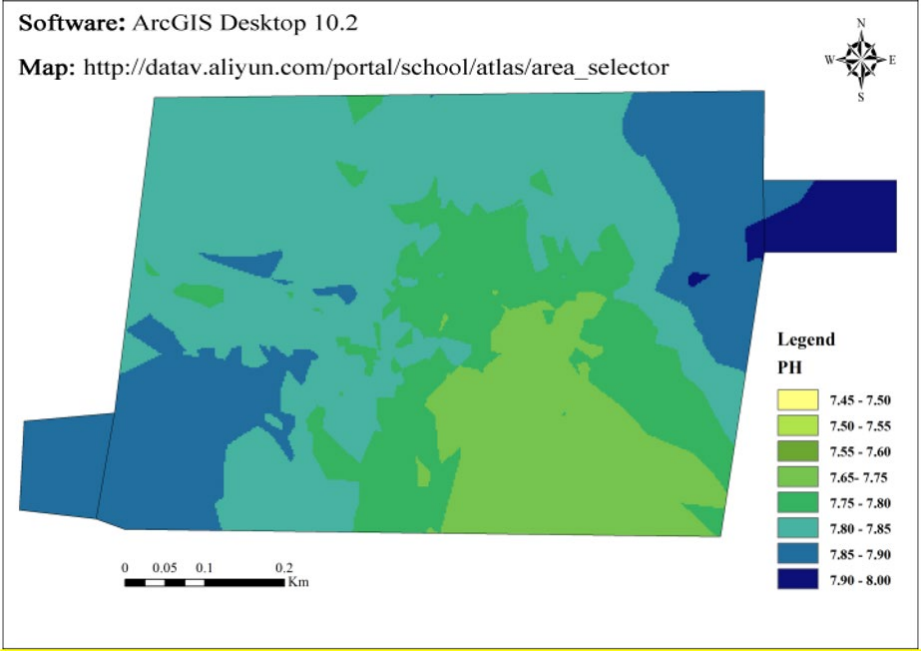
Spatial distribution of soil pH in sampling area.

According to Fig. 3, the soil pH in the study area ranges from 7.50 to 8.18, with a mean of 7.82, regarding the *Specification of land quality geochemical assessment* (DZ/T 0295-2016)^44^ for the definition of soil acidity, the Kriging interpolation analysis of soil acidity is conducted by using ArcGIS, and the results are shown in Fig. 4. All the soil samples are alkaline, and the soil in the study area was mainly alkaline. When the soil environment where the heavy metal pollutants are located is alkaline, the heavy metals adsorbed on the soil colloids are easy to combine with the hydroxide ion in the alkaline soil to form stable compounds. Heavy metals' dissolution rate and solubility decreased, making the heavy metals in the soil not easy to migrate and deposit^45^. When plants absorb heavy metals, they will cause a decline in crop yield and quality and endanger human health through the food chain.

#### Statistical description of heavy metal content

Table 4 is a descriptive statistical analysis of heavy metals in the Anxin County and lists the statistical results of the contents of five heavy metals of Cu, Zn, Pb, Cd, and Ni in the study area. The average contents of Cu, Zn, Pb, Cd, and Ni are 63.73 ± 11.74, 171.66 ± 28.79, 136.69 ± 51.07, 0.73 ± 0.25 and 41.43 ± 2.81 mg·kg^−1^, respectively. The average values of Cu, Zn, Pb, Cd, and Ni are 2.92 times, 2.19 times, 6.36 times, 7.78 times, and 1.34 times of the background values of soil elements in Hebei, respectively. The contents of Cu, Zn, and Ni in the soil are all less than the risk screening value, and the risk to the quality and safety of agricultural products, crop growth, or soil ecological environment is low and can be ignored in general. The soil Pb and Cd content percentages between the risk screening value and risk intervention value are 16.67% and 64.58%, respectively. The problem of exceeding the standard of Pb and Cd is more serious, and there may be risks to the quality and safety of agricultural products, crop growth, or soil ecological environment, and safe utilization measures should be taken.

**Table 4:** Statistical analysis of soil lead and cadmium in Anxin County.

Element	Cu	Zn	Pb	Cd	Ni
Maximum (mg kg^−1^)	92.34	266.40	336.00	1.57	45.65
Minimum (mg kg^−1^)	41.57	134.39	65.00	0.35	30.46
Average value (mg kg^−1^)	63.73	171.66	136.69	0.73	41.43
Standard deviation	11.74	28.79	51.07	0.25	2.81
Coefficient of variation	0.18	0.17	0.37	0.35	0.07
Mid-value (mg kg^−1^)	63.62	168.37	129.00	0.72	41.70
Kurtosis	− 0.39	0.94	4.57	0.89	3.40
Skewness	0.10	0.97	1.77	0.77	− 1.29
P-value	0.200	0.174	0.09	0.200	0.034
Background value (mg kg^−1^)	21.8	78.4	21.5	0.094	30.8
Risk screening value (A) (mg kg^−1^)	100	300	170	0.6	190
Risk intervention value (B) (mg kg^−1^)	–	–	1000	4	–
Above A and below B (%)	0	0	16.67	64.58	0
Above B (%)	0	0	0	0	0

The coefficient of variation (CV) is the ratio of standard deviation to the average value, which can characterize the degree of dispersion of data. The CV is classified into a weak variation (CV < 0.1), moderate variation (0.1 < CV < 0.9), or high variation (CV > 0.9)^46^. By analyzing the characteristics of heavy metal content in the study area, it can be seen that the distribution of the five elements in the local soil is moderately variable, and the CV of Pb and Cd is large. It shows that the distribution of Pb and Cd is uneven, and it is affected to some extent by human activities.

It can be seen from Table 4 that the kurtosis and skewness of Cu and Cd are closer to 0. The K-S test shows that the two-sided significance of Cu, Zn, Pb, and Cd are all greater than 0.05, and the data of the four heavy metals obey the normal distribution. The two-sided significance of Ni is less than 0.05, and there is a significant deviation in the data distribution, which does not obey the normal distribution.

#### Level distribution characteristics of soil heavy metals

**Figure 5:**
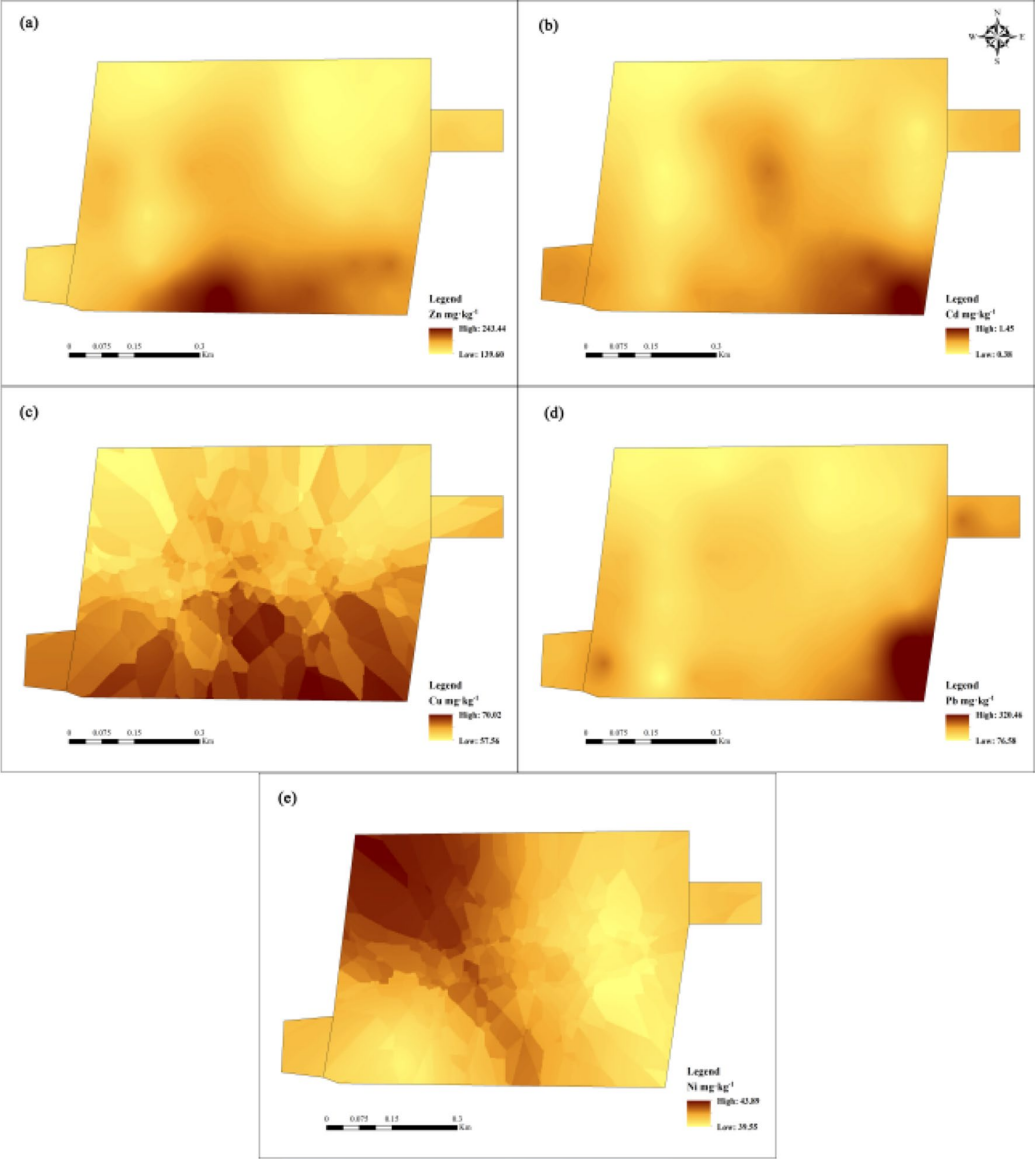
Spatial distribution of heavy metal elements in soil of the study area (**a**) Zn (**b**) Cd (**c**) Cu (**d**) Pb (**e**) Ni.

Figure 5 is a spatial analysis of the distribution of different heavy metal elements in the soil of the study area. The results show that Zn shows a high-concentration band distribution in the south of the study area; Cu shows a large-scale block distribution in the study area; Cd and Pb show a similar distribution trend in the study area, with high concentration areas in the southeast of the study area; For Ni, the concentration is higher in the northwest of the study area. According to Figs. 2 and 5, it can be seen that the farmland soils in the study area have generally high levels of heavy metals, and Pb and Cd pollution is severe. It is related to the presence of metal-smelting factories in the surroundings. The extensive industrial model is the primary source of heavy metal pollution in the surrounding soils. At the same time, the large traffic flow in the study area, vehicle exhaust emissions, oil leakage, and rubber tire wear may also be important reasons for the enrichment of heavy metals such as Cd and Pb in the soil in this area^47^.

#### Characteristics of Cd and Pb content in soil profile

**Figure 6:**
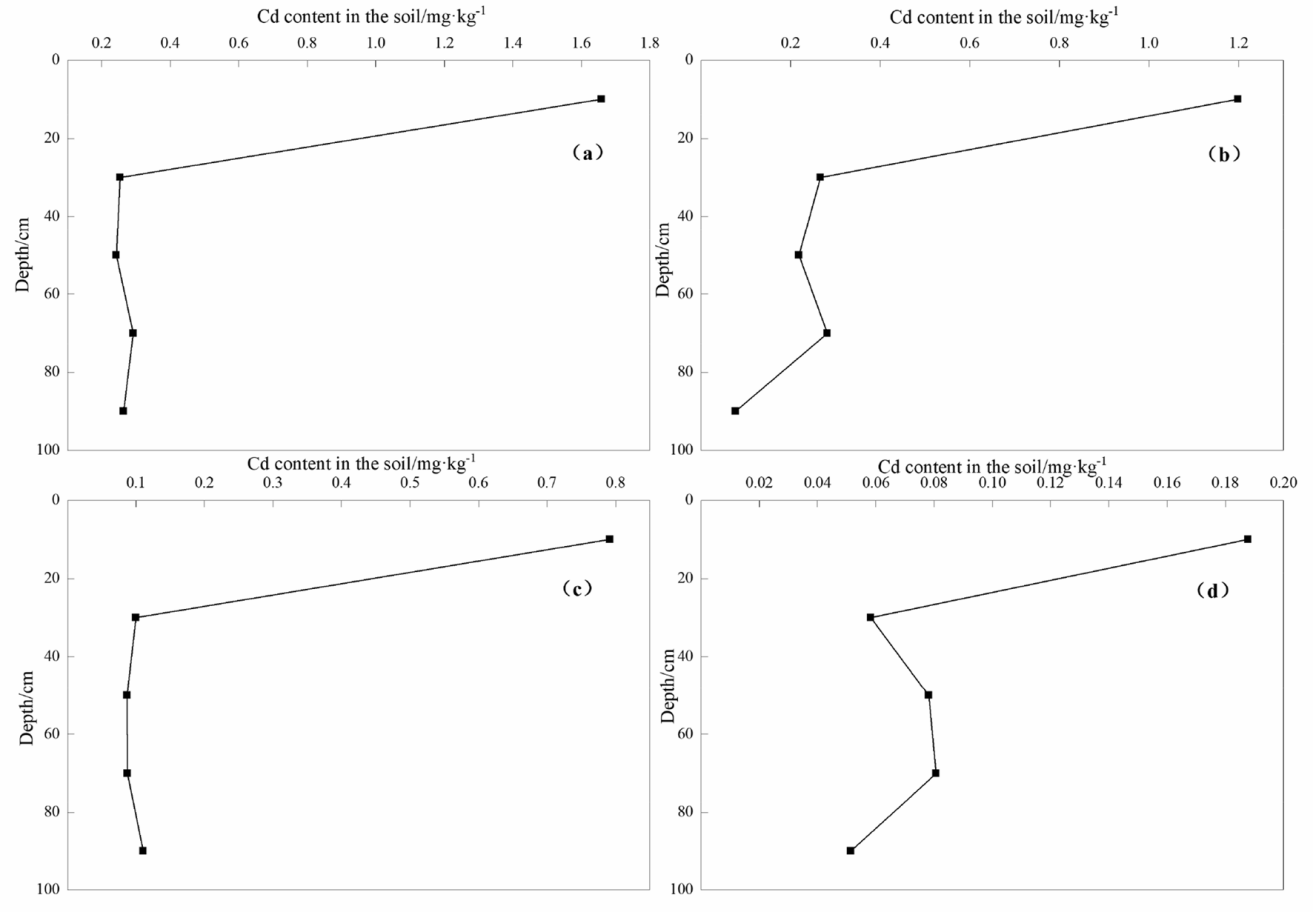
Cd concentrations at different depth in soil profiles. (**a**) D3 soil profile (**b**) D2 soil profile (**c**) D1 soil profile (**d**) D0 soil profile.

**Figure 7:**
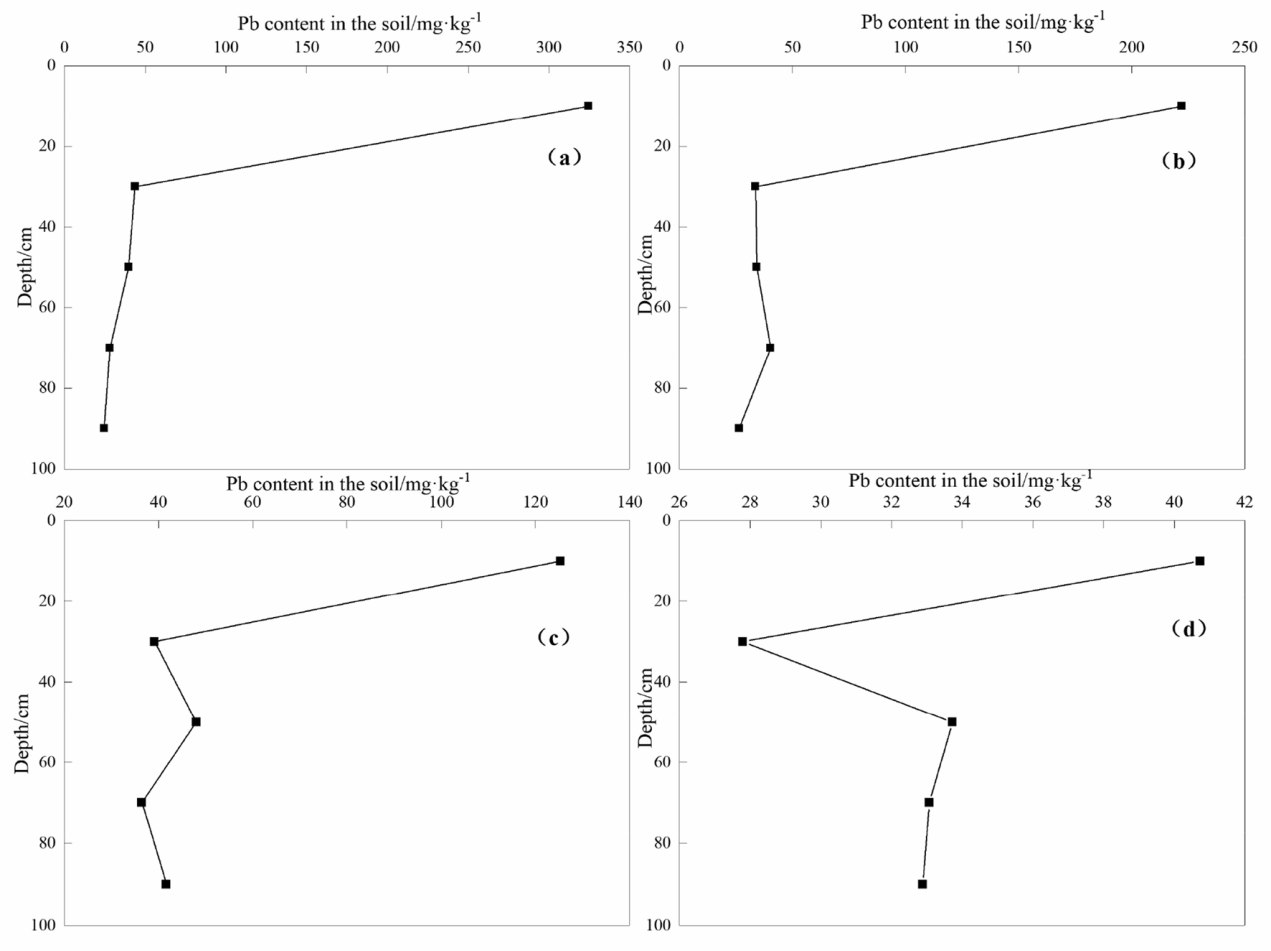
Pb concentrations at different depth in soil profiles. (**a**) D3 soil profile (**b**) D2 soil profile (**c**) D1 soil profile (**d**) D0 soil profile.

In the vertical section of the soil, the distribution of elements is not only restricted by its geochemical properties, soil-forming parent material, and soil-forming processes under natural conditions^48^ but also closely related to human activities^49^. The same element may have multiple distribution types in different sections of soil profiles, but the probability of a specific type is relatively high. In the four soil profiles of D0, D1, D2, and D3 studied in this work, the vertical distribution characteristics of heavy metals Cd and Pb on the profiles have both commonalities and differences. According to Figs. 6 and 7. The heavy metal cadmium and lead in the soil is mainly distributed in the 0–20 cm of the soil surface. As the depth increases, the content of elements that migrate to a depth below 20 cm is significantly reduced, and the content of cadmium and lead generally appears to become smaller from shallow to deep. Trends and changes are relatively large. The enrichment of elements in the surface soil may be related to the fact that the farmland has been affected by the surrounding human activities^50^, including perennial agricultural farming, road transportation, and the production activities of the surrounding heavy metal processing industry. These factors have significant effects on the vertical migration and enrichment of heavy metals in the soil. The vertical distribution trends of heavy metal Cd and Pb elements in the four soil profiles are the same, but the contents in the 0–20 cm layer are different. The main reason is that the four soil profiles are located in different blocks. D1, D2, and D3 are heavy metal-contaminated farmland soils in the study area.

### Availability characteristics of heavy metals in farmland soil

#### The available content of heavy metals in soil

Table 5 lists the statistical results of the practical content of the two primary polluting heavy metals, Cd and Pb, in the study area. The practical content of Cd and Pb ranges from 0.22 to 1.15 mg kg^−1^ and 10.00 to 75.10 mg kg^−1^, respectively, and the average effective content of Cd and Pb is 0.47 ± 0.18 mg kg^−1^ and 28.69 ± 13.75 mg kg^−1^, respectively. By analyzing the dispersion degree of the available content of Cd and Pb in the study area, it can be seen that the distribution of Cd and Pb in the local soil is moderately variable. At the same time, the coefficient of variation of Pb is more significant, indicating that Pb is affected by human activities to a certain extent. The effect is more vital than Cd.

**Table 5:** Analysis of the available content of Cd and Pb in the soil in Anxin County.

Element	Cd	Pb
Maximum (mg kg^−1^)	0.09	1.03
Minimum (mg kg^−1^)	0.02	0.02
Average value (mg kg^−1^)	0.04	0.28
Standard deviation	0.02	0.22
Coefficient of variation	0.46	0.79
Mid-value (mg kg^−1^)	0.03	0.21

#### Heavy metal element availability index

**Figure 8:**
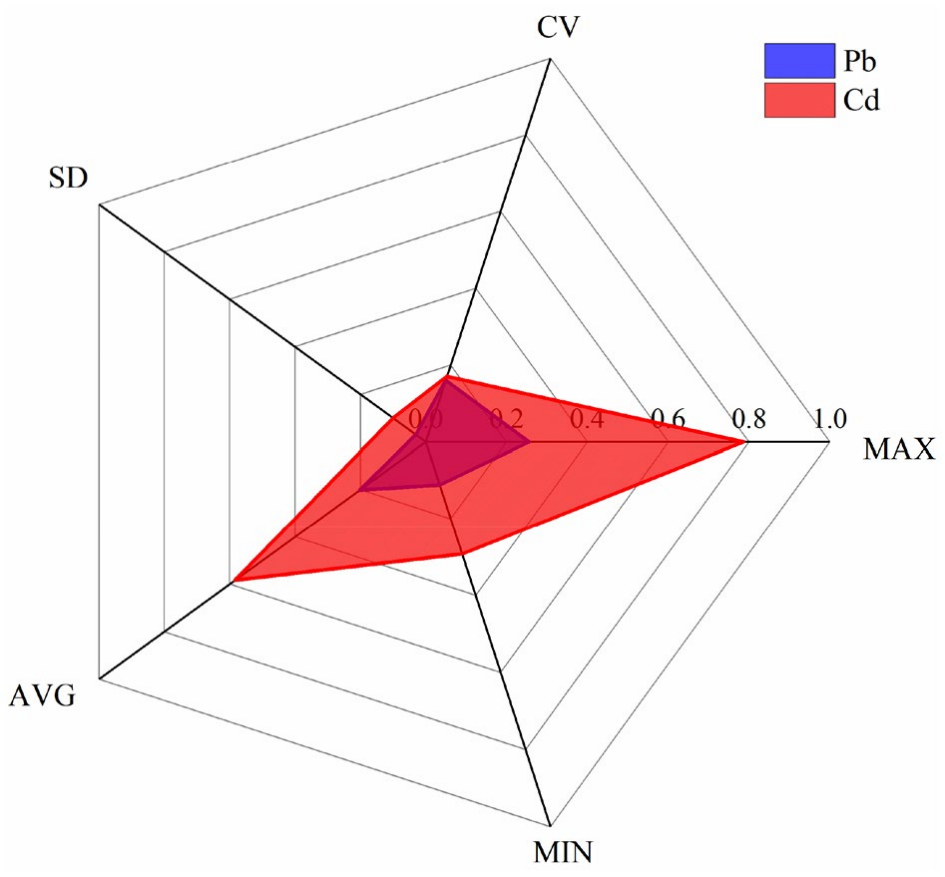
Analysis results of activation rate of lead and cadmium in soil.

The heavy metal element availability index is the ratio of the applicable state content of an element to the total amount of this element in the sample. The activity index can reflect soil's current and potential supply levels^51^. The study area is mainly dominated by Cd and Pb pollution, so the practical content of Cd and Pb at 48 sites in the study area is analyzed (Fig. 8). The results show that the availability index of the two heavy metal elements is quite different. The mean values of Cd and Pb availability indices are 58.42% and 20.26%, respectively, and the soil Cd availability index is higher than other elements. Relevant studies have shown that applying calcium fertilizer in weak alkaline soil can reduce the availability index of soil Cd^52^.

### Characteristics of heavy metal content in wheat

Table 6 lists the statistical results of Cd and Pb content in wheat grains in the study area. The contents of Cd and Pb varied in the range of 0.02–0.09 mg kg^−1^ and 0.02–1.03 mg kg^−1^, respectively, and the average contents of Cd and Pb were 0.04 ± 0.02 mg kg^−1^ and 0.28 ± 0.22 mg kg^−1^, respectively. By analyzing the dispersion degree of Cd and Pb content in wheat grains in the study area, it can be seen that the distribution of Cd and Pb in wheat grains is moderately variable, while the coefficient of variation of Pb is closer to 1, indicating that Pb is affected by soil to a certain extent. The change of Pb content has a significant influence. *National Food Safety Standard—Limits of Contaminants in Foods* (GB2762-2017)^53^ stipulates that the limit values of Cd and Pb in wheat grains are 0.1 mg kg^−1^ and 0.2 mg kg^−1^, respectively. Among the 48 wheat samples investigated, the Cd content meets the standard, but 4.17% of the samples are close to 0.1 mg kg^−1^ (more than 0.09 mg kg^−1^). The content of Pb in 50% of the samples exceeds the standard, and the Pb in the wheat grains exceed the standard seriously.

**Table 6:** Statistics of Cd and Pb content in wheat grain.

Element	Cd	Pb
Maximum (mg kg^−1^)	0.09	1.03
Minimum (mg kg^−1^)	0.02	0.02
Average value (mg kg^−1^)	0.04	0.28
Standard deviation	0.02	0.22
Coefficient of variation	0.46	0.79
Mid-value (mg kg^−1^)	0.03	0.21
Limits of heavy metals in food (mg kg^−1^)	0.1	0.2
Proportion of samples exceeding the limit of heavy metals in food (%)	0	50

### Evaluation results of heavy metal pollution in soil

#### Evaluation results of single pollution index method

**Figure 9:**
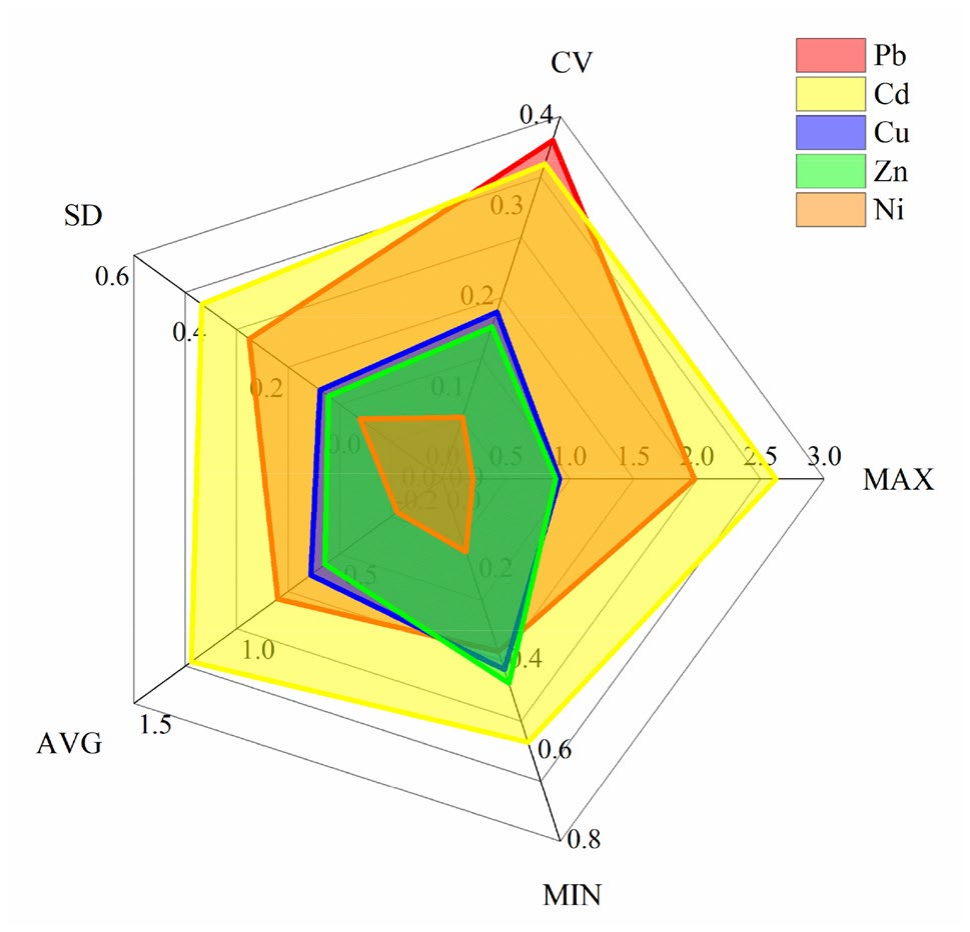
Evaluation results of single factor pollution index method.

The evaluation of five heavy metal elements by the single pollution index method is shown in Fig. 9, and it can be concluded that the soil heavy metal pollution in the study area is Cd, Pb, Cu, Zn, and Ni in order. The pollution degree of Cd is relatively severe; the average value of the pollution index is 1.22, the variation range is 0.58 ~ 2.62, and the CV is 0.35. Followed by Pb pollution, the average value of the pollution index is 0.80, the range of change the variation range is 0.38 ~ 1.98, and the CV is 0.37. The maximum single pollution index of Cu, Zn, and Ni did not exceed 1, indicating that Cu, Zn, and Ni slightly polluted the sampling area. Field investigation found many small-scale non-ferrous metal recovery and smelting plants around the study area, and many trucks carrying scrap cables and metals pass by the eastern side of the research area. Many Cd and Pb-containing substances from factory production and automobile exhaust entered the soil through atmospheric deposition and waste residue infiltration^54^.

#### Evaluation results of potential ecological risk index method

**Figure 10:**
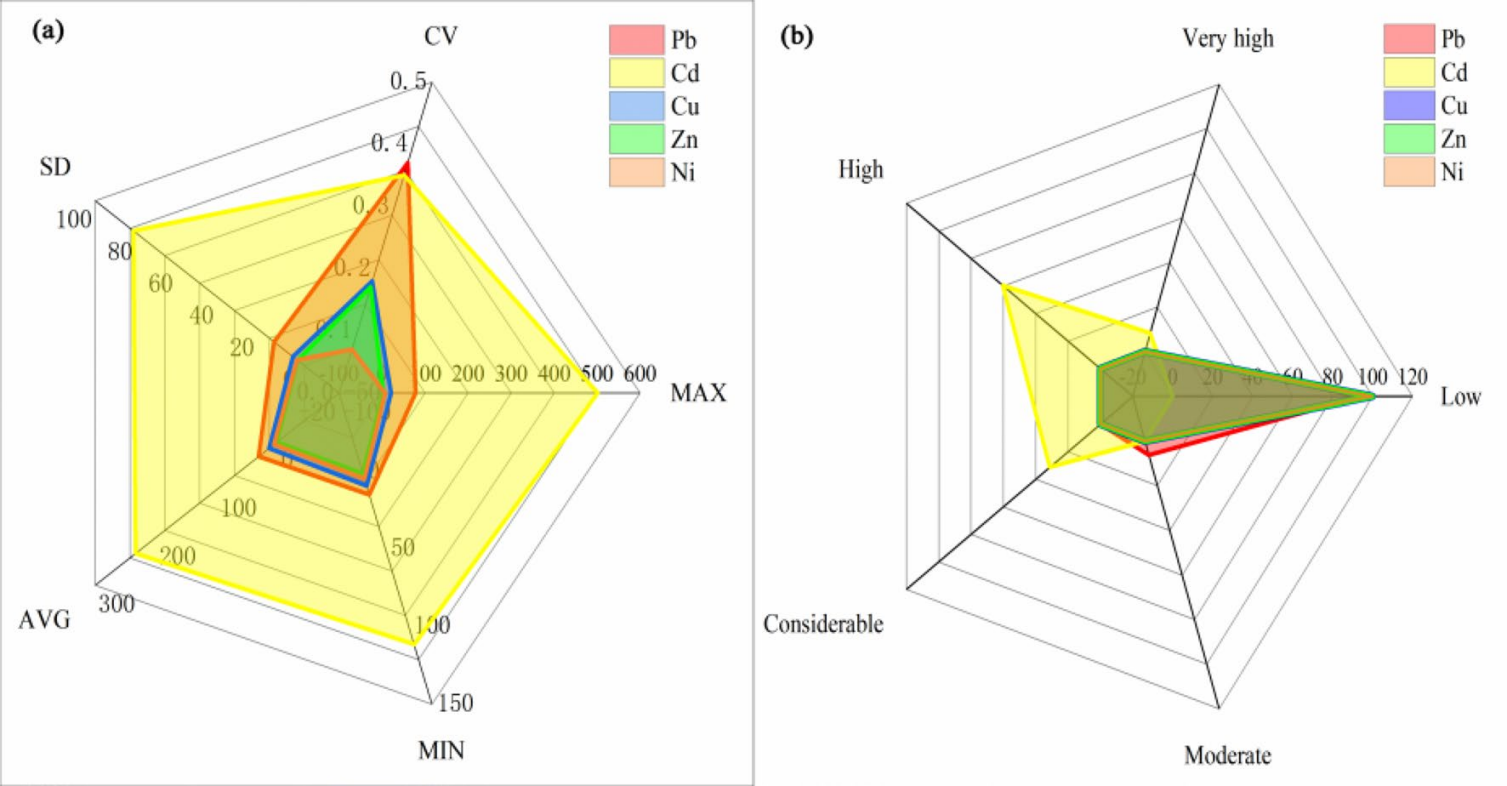
Statistical analysis of single factor ecological risk index. (**a**) The single factor ecological risk index of five heavy metals. (**b**) Evaluation results of the single factor ecological risk index of five heavy metals.

**Figure 11:**
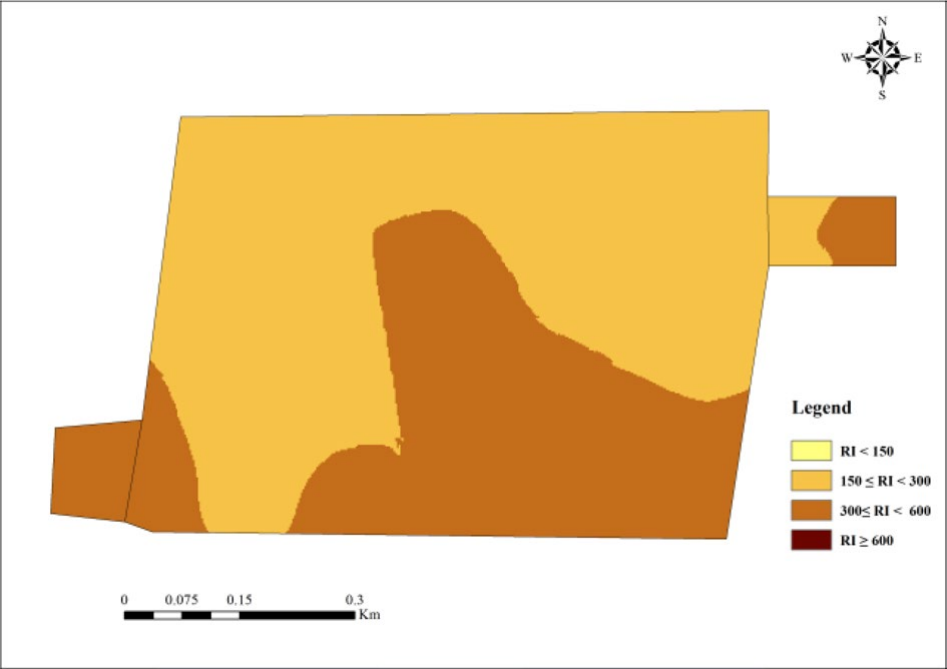
Evaluation results of potential ecological risk index method.

Figures 10 and 11 are the evaluation results of heavy soil metals' potential ecological risk index. Except for Cd, the average value of the single factor ecological risk index of the other four heavy metal elements is less than 40, which belongs to low ecological risk. The order of the five heavy metals in the study area from the largest to the smallest is Cd > Pb > Cu > Zn > Ni, Cd and Pb are seriously polluted in this area. The Cd and Pb single-factor ecological risk indexes CV are 0.35 and 0.37, respectively, higher than those of the other three heavy metals, indicating that Cd and Pb are unevenly distributed in the soil in the study area are greatly affected by human activities. The average value of Cd’s single factor ecological risk index is 233.51, which belongs to high ecological risk. The proportions of the single factor ecological risk index reaching consultable, high, and very high are 31.25%, 60.42%, and 8.33%, respectively.

The potential ecological risk index method (Fig. 11) shows that the higher ecological risk is distributed in the east and central-southern study area. 56.25% of the samples are of moderate hazard level, and the samples with considerable hazards accounted for 43.75%. Pollution may be related to the "three wastes" produced by non-ferrous metal smelting in the study area and the exhaust gas produced by vehicles on traffic roads. Heavy metals such as Cd and Pb enter the surrounding farmland soil in different ways and accumulate continuously^55^.

#### Evaluation results of geoaccumulation index method

**Figure 12:**
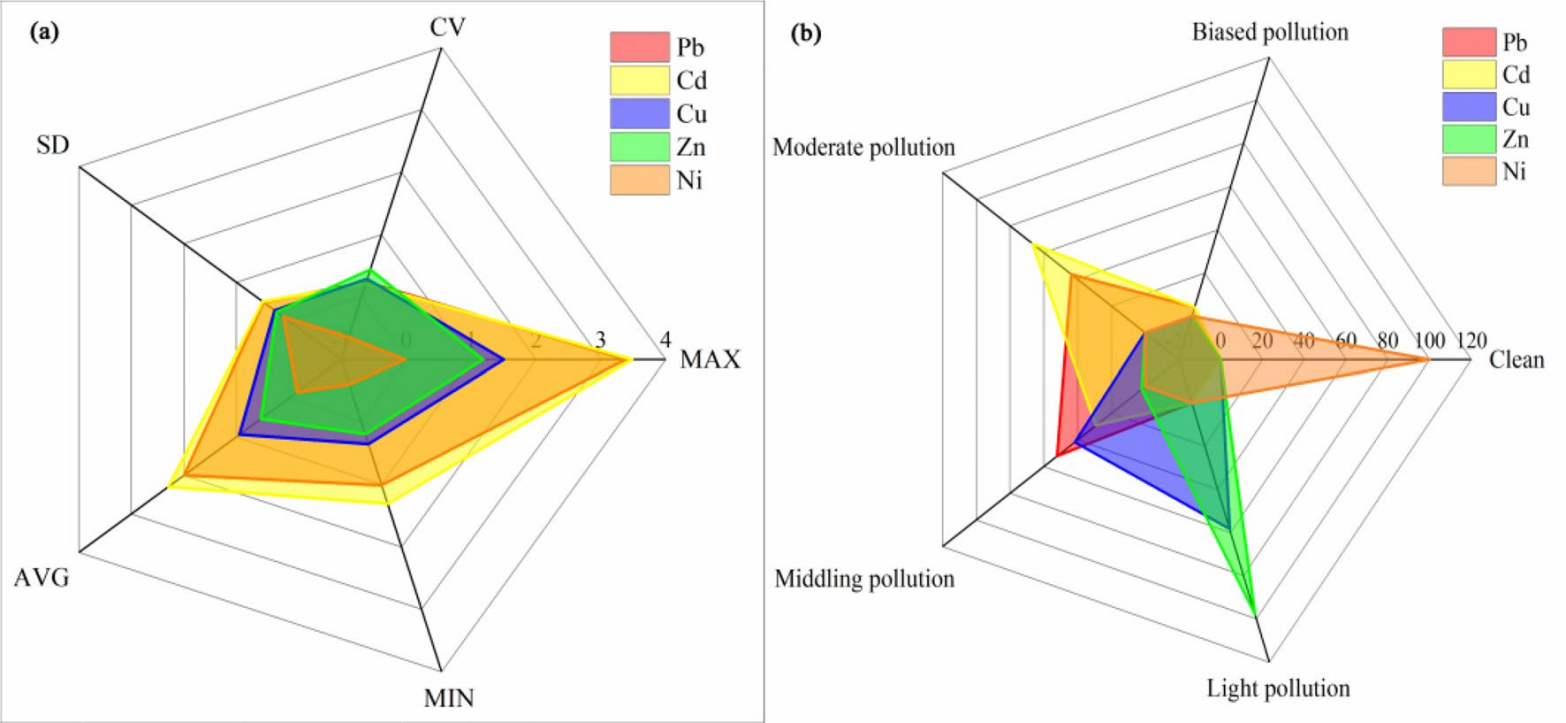
Statistical analysis of heavy metal geoaccumulation index in soil. (**a**) The geoaccumulation index of five heavy metals. (**b**) Evaluation results of the geoaccumulation index of five heavy metals.

According to Fig. 12, it can be seen that the geoaccumulation index of Ni in the study area is less than 0, which is a pollution-free level; The average values of the Cu and Zn geoaccumulation indexes are 0.94 and 0.53, which are at a light pollution level; The geoaccumulation index of Pb is 2.00, which is at a middling pollution level, and the geoaccumulation index of Cd is 2.29, which is at a moderate pollution level.

**Figure 13:**
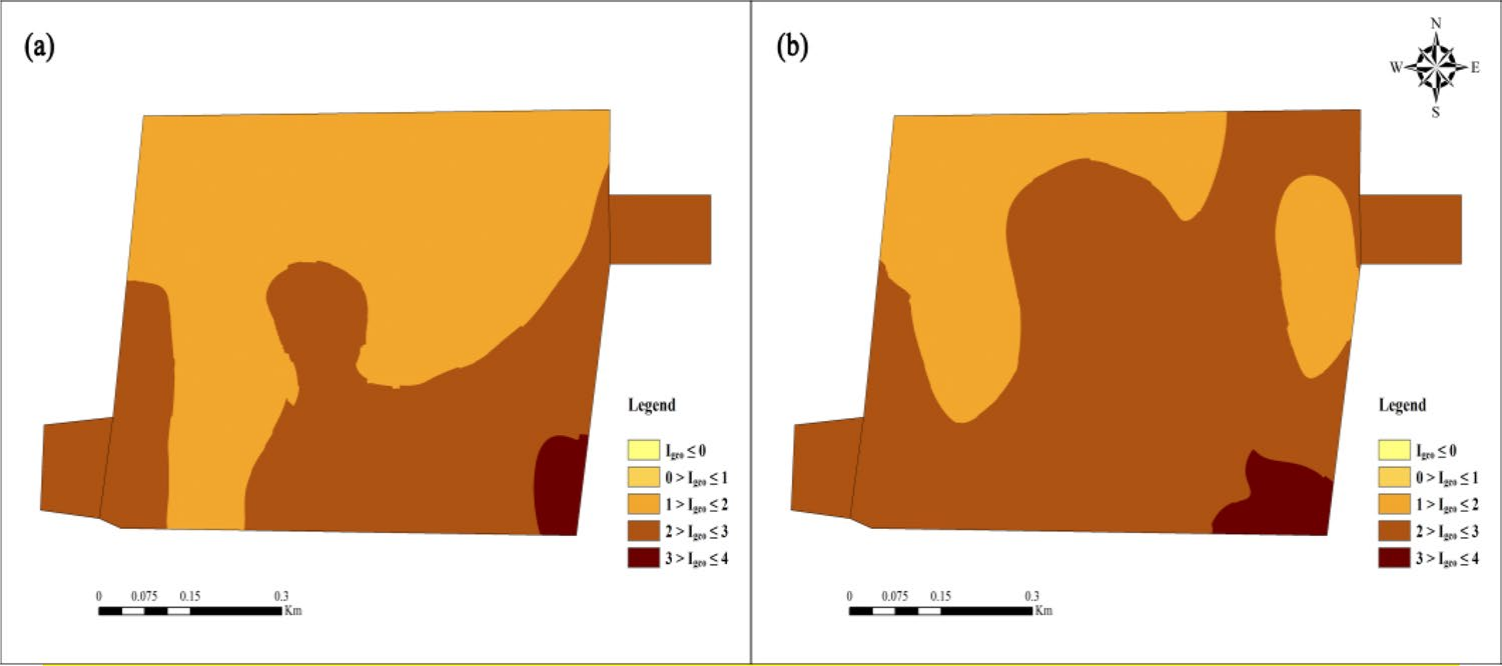
Evaluation results of geoaccumulation index method (**a**) Pb (**b**) Cd.

The proportion of soil sample pollution classification shows no Ni pollution in the study area, and the pollution rates of the four elements of Cu, Zn, Pb, and Cd all reach 100%. Among them, for Pb, 52.08% of the soil is at a middling pollution level, 43.75% of the soil is at a moderate pollution level, and 4.17% of the soil is at a biased pollution level; For Cd, 29.17% of the soil is at a middling pollution level, 66.67% of the soil is at a moderate pollution level, and 4.17% of the soil is at a biased pollution level; For Cu, 58.33% of the soil is at a light pollution level, and 41.67% of the soil is at a middling pollution level; For Zn, 97.92% of the soil is at a light pollution level, and only 2.08% of the soil is at a middling pollution level. Cu and Zn contribute less to soil pollution, while Pb and Cd contribute the most. The significant standard deviation of Pb and Cd indicates that the Pb and Cd have a high degree of dispersion in the geoaccumulation index and a high variation coefficient. The two elements in the soil are significantly interfered with by the production activities of heavy metal processing enterprises around the farmland, which leads to severe pollution of Pb and Cd in the local soil. According to the evaluation results of the geoaccumulation index (Fig. 13), it can be concluded that the areas where the soil is at Pb moderate-biased pollution level are mainly distributed in the southeast and southwest of the study area; the farmland near the road in the southeast of the study area have Cd moderate-biased pollution levels in the soil. It is affected by the waste non-ferrous metal recycling, electrolysis, and other related production activities of surrounding enterprises and the exhaust gas generated by the driving of vehicles.

### Principal component analysis

**Figure 14:**
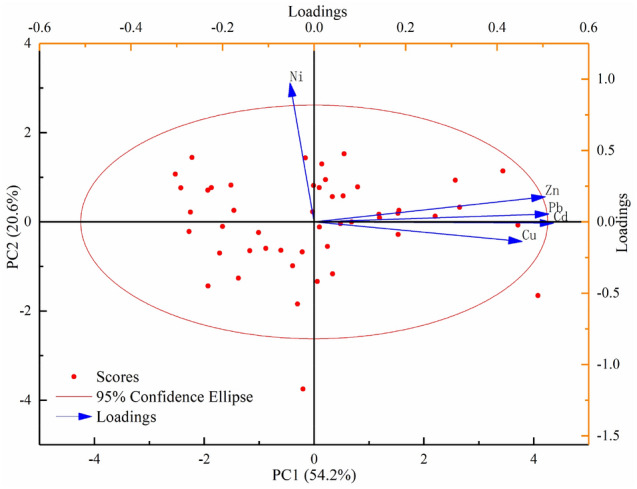
Principal component analysis on factor loading.

The principal component analysis is a multivariate statistical analysis method that converts multiple indicators into a few unrelated comprehensive indicators and classifies the comprehensive indicators according to specific rules. It is often used to identify the source of elements in the medium and obtain the contribution rate of different sources to the same element^56–58^. Using SPSS software to perform KMO test on the data, the obtained statistic value is 0.742, and the accompanying probability of the Bartlett sphericity test is 0.000. Therefore, principal component analysis and discriminant classification techniques can be used to study pollution types of farmland around the heavy metals industry in Anxin County, Hebei Province of China, and the analysis results are shown in Table 7 and Fig. 14. Carrying out Varimax orthogonal rotation on the Kaiser normalized factors, two principal components with eigenvalues greater than one are obtained, with variance contribution rates of 54.033% and 20.678%, respectively, and the cumulative contribution rate of 74.711%. It can explain most of the information of heavy metal elements in the soil of the researched area.

**Table 7:** Matrix analysis matrix of soil heavy metals in the researched area.

Element	PC1	PC2
Cu	0.738	− 0.184
Zn	0.840	0.128
Pb	0.845	0.005
Cd	0.859	− 0.060
Ni	− 0.027	0.990
Initial eigenvalue	2.702	1.034
Variance contribution (%)	54.033	20.678
Cumulative variance contribution rate (%)	54.033	74.711

Cd, Pb, Zn, and Cu show higher loads on the first principal component, respectively 0.859, 0.845, 0.840, and 0.738. The four elements are significant within the 95% confidence interval, indicating that the four elements have the same pollution source. The spatial distribution of the four heavy metal elements is relatively similar. The areas with higher content are concentrated in the southeast of the study area, the distribution area is close to the road, and there is a heavy metal processing plant area around. The average value of the four heavy metal elements is higher than the soil background value, indicating that it is significantly affected by human activities. Related research shows that metal smelting and other production activities will increase the content of Cd in the atmosphere, Cd sedimentation causes pollution to the soil^59^, and the accumulation of industrial waste is also an essential source of Cd pollution^60,61^. Most cars use leaded gasoline as fuel, and long-term use has caused the accumulation of soil Pb^62,63^. Because of its high corrosion resistance and high thermal conductivity, Cu is an essential component of vehicle braking systems and automotive radiators^64^. The wear of automobile parts will cause copper to enter the surrounding environment, so It is also often used as the identification element of the source of traffic^65,66^. Maximilian et al.^67^ found that Zn can also be a marker element for mobile sources. Cu, Pb, and Zn will enter the surrounding environment as the car parts wear out during the driving process^68,69^. PC1 is closely related to Cd, Zn, Cu, and Pb and has the characteristics of a traffic source and an industrial source. Therefore, it is speculated that PC1 may be a traffic source and an industrial source, mainly affected by vehicle traffic, exhaust emissions, and industrial production.

Ni exhibits a higher load on the second principal component, which is 0.990. The Ni in the soil mainly comes from rock weathering, atmospheric dust reduction, irrigation water (including nickel-containing wastewater), farmland fertilization, and the decay of plant and animal residues^58,70^. The evaluation result of the soil accumulation index of Ni is pollution-free, and the contribution of human activities to Ni is relatively low, mainly natural sources^71^. The content is mainly affected by the soil-forming parent material. Therefore, it is speculated that the pollution sources represented by PC2 are natural sources and agricultural sources.

### Comparison of heavy metal pollution in farmland soil in different regions

In order to better analyze the pollution characteristics of heavy metals in different types of farmland soils, the results of this study were compared with the average values of heavy metal contents obtained by other studies. The comparison results are shown in Table 8. Compared with foreign studies, the concentration of Cd and Pb in this study area is higher, which may be related to the different industrial structures. For example, fertilizers containing Cd are not used in Kerman. Shanxi Province has a long history of gold mining and smelting, and the management system is not standardized. The random stacking of tailings slag from the gold mining and smelting process is the source of heavy metal pollution in the soil of nearby farmland^78^. Compared with the study by Zhou et al.^73^ in Hebei Province, the average content of Cd and Pb is slightly lower, but the difference is not significant because the selected study area is different, and there are differences in human interference. Due to the different soil properties in different plots, the results obtained are also different, but overall, there is serious Cd and Pb pollution. The results of this study are consistent with those of previous studies.

**Table 8:** Comparison of heavy metal pollution in farmland soil in different regions (mg kg^−1^).

Area	Heavy metal pollution	Average Cd content	Average Pb content
This research	Cd > Pb > Cu > Zn > Ni	0.73	136.7
Around the Pyrite Mine Area in Zhejiang Province^72^	Cd > Cu > Pb > Zn	0.53	73.0
Around Hebei Metal Smelting Enterprises^73^	Cd > Pb > Ni > Zn	1.42	158.7
Around the Gold Mine Area in Shaanxi Province^74^	Pb > Cd > Cu > Zn > Ni	2.91	1114.1
Sruwen Tengaran Semarang-Indonesia^75^	Cd > Pb > Cu	2.00	30.0
Kurdistan Region-Iraq^76^	Co > Cd > Cr > Pb	0.70	18.6
Kerman, Iran^77^	Hg > Cd > Pb > Cu > Ni	0.16	39.1

## Conclusion

In summary, this paper reviews the heavy metal pollution of farmland soil in Anxin County, Hebei, China. The main points obtained through the analysis are as follows:

The soil in the study area is weakly alkaline, and the contents of Cu, Zn, and Ni in the soil are all lower than the risk screening values for soil contamination of agricultural land. In comparison, the contents of Cd and Pb are higher than the risk screening values for soil contamination of agricultural land and lower than the risk intervention values for soil contamination of agricultural land. The proportions of the values points are 64.58% and 16.67%, respectively, and the pollution is mainly concentrated in the topsoil of 0–20 cm. According to the *National Food Safety Standard—Limits of Contaminants in Foods* (GB2762-2017), the Cd content in wheat grains all meets the standard, and the Pb content in 50% of the wheat grain samples exceeds the standard lead limit. There is a risk of Cd and Pb contamination in the study area.

The evaluation results of the single pollution index, potential ecological risk index, and geoaccumulation index show that the order of soil heavy metal pollution risk in the study area is Cd > Pb > Cu > Zn > Ni. The potential ecological risk index in the study area is 288.83, and the soil heavy metal pollution is at a moderate-considerable ecological risk level. The average value of Cd's single-factor environmental risk index is 233.51, which belongs to the high environmental risk and is the main influencing factor. Pb and Cd pollution is more severe than the other elements, and the heavily polluted areas are mainly distributed in the southeast of the study area. Cd and Pb in soil are significantly disturbed by the production activities of heavy metal processing enterprises around the farmland.

Principal component analysis/absolute principal component score (PCA/APCS) receptor model analysis results show two primary soil heavy metal pollution sources in the study area. It is speculated that source 1 may be industrial and mobile sources, and source 2 may be agricultural and natural sources. Cd, Pb, Zn, and Cu are mainly industrial and mobile sources, and the sources of Ni are mainly agricultural and natural sources.

While building Anxin County, environmental management and restoration must be done well. Focus on strengthening the attention to Pb and Cd in the soil of the Anxin County while carrying out soil remediation work and doing an excellent job of prevention and control, urging heavy metal processing enterprises to improve production processes, shutting down some heavily polluting enterprises to avoid developing in a more serious direction. In the future construction process, soil restoration is still an arduous task.

## Data Availability

The datasets used and/or analysed during the current study available from the corresponding author on reasonable request.
